# “Hurt and Misunderstood”: Emotional reactions to pain dismissal in emerging adults with chronic pain

**DOI:** 10.1016/j.hctj.2025.100116

**Published:** 2025-08-04

**Authors:** Elizabeth Fenelon, Kayla McCracken, Keely Bieniak-Fortier, Chloe Crosby, Paulina Paredes Cienega, Susan T. Tran

**Affiliations:** DePaul University, 2219 North Kenmore Avenue, Chicago, IL 60614, United States

**Keywords:** Pain dismissal, Emerging adults, Chronic pain, Gender, Ethnicity

## Abstract

**Background:**

Twelve percent of emerging adults (EAs) experience chronic pain which is associated with psychological distress and isolation. At least 40 % of EAs with chronic pain experience pain dismissal which poses a significant barrier to care and treatment. The goals of this paper were to expand our understanding of pain dismissal experiences among EAs and to extend research to the emotional and psychological impact of pain dismissal on EAs.

**Method:**

EAs with chronic pain (*N* = 227) between the ages of 18–25 (*M*age = 19.58) completed online surveys of pain experiences, anxiety, and depression. Thematic analysis was conducted for three open-ended responses.

**Results:**

Over 40 % of EAs with chronic pain experienced pain dismissal (43 %), with female and EAs identifying as other gender identity experiencing greater dismissal than male EAs. There were no differences across racial/ethnic identities. *Medical professionals* (46 %) and *caregivers/parents* (38 %) were most frequently reported people to have dismissed participant pain. *Minimizing/invalidating* (27 %) and *normalizing* (26 %) were the most frequent themes of what was said to dismiss one’s pain. In response to pain dismissal, EAs described feeling *gener*ic *negative feelings* (25 %) and *anger/annoyance* (21 %).

**Discussion:**

Survey responses suggest that pain dismissal bothered EAs, and those who experienced pain dismissal had higher anxiety and depression, indicating a need for family/provider education on pain in EAs and validation of pain experiences in EAs. New themes around what constituted pain dismissal included *negative self-view* and *invalidated*/*gaslit*. Future research should explore the long-term effects pain dismissal has on chronic pain outcomes.

## 1 Introduction

Emerging adulthood (EA) is a developmental period occurring between 18 and 25 years of age marked by increased independence, self-exploration, and psychosocial adjustment^1^. Recurrent pain is experienced by approximately 12 % of EAs^2^. Chronic pain can last years, including from childhood through to adulthood^3^. Across developmental phases, chronic pain is associated with significant psychosocial impacts, including psychological distress^4^, ^5^, ^6^ and social isolation^7^, ^8^. Developmentally, EAs are already facing numerous changes including increasing independence, social changes, and economic responsibility^9^, ^10^, ^11^, ^12^; and COVID-19 has disrupted developmental trajectories of EAs^13^. Thus, EAs with chronic pain face these developmental milestones amidst added physical, psychosocial, and economic strain.

Individuals seeking care for pain are ultimately hoping for their pain to be recognized by care providers and to find treatment^14^. Unfortunately, at least 40 % of EAs with chronic pain experience pain dismissal^15^, ^16^. Pain dismissal, which is expressing disbelief of, ignoring, or diminishing someone's pain, is a significant barrier to care; having a provider dismiss one’s pain may discourage or prevent a patient from receiving further treatment. Thus, increasing our understanding of pain dismissal experiences of EAs with chronic pain is essential to determine how to diminish disability and difficulty across future developmental phases. Additionally, research into pain dismissal is necessary when considering the health care transitions of emerging adults with chronic pain. For many individuals with pediatric chronic pain, this experience can extend into adulthood; therefore, youth must transition from pediatric health care services to adult health care services. This can be a challenging time for emerging adults especially, since these individuals are already experiencing emotional turbulence in their lives due to gaining independence. Emerging adults with chronic pain can experience stress along with daily hassles that affect psychosocial functioning; it is necessary to help make their health care transition as easy as possible because of these factors^17^. The experience of pain dismissal within a health care setting can be deleterious for these emerging adults with chronic pain, as felt stigma is a barrier to adequate health care and potential contributor to health inequities^18^, ^19^. There is limited research on pain dismissal in EAs; thus, it would be beneficial to investigate whether previous findings can be replicated and whether individual characteristics such as race or gender are related to pain dismissal^20^. Previous research found that 43 % of EAs with chronic pain experienced pain dismissal. Pain dismissal was reported more frequently by female EAs (70 %) compared to male EAs (29.7 %). Those that dismissed the pain of these EAs included parents (52 %) and physicians (30 %). Unfortunately, physicians often feel inadequately prepared to treat chronic pain, which can then result in delayed treatment or no treatment at all^21^. Other categories of individuals that dismissed the pain of EAs included friends (28 %), teachers (25 %), siblings (21 %), peers (19 %), and coaches (16 %).

Pain dismissal can be conveyed in various ways. Defenderfer and colleagues identified themes in the responses of EAs who reported what was said when they felt dismissed. The most common category of pain dismissal was denial or disbelief, which occurred 39 % of the time. Other categories of pain dismissal included minimizing pain (diminishing the pain experience), faking it (states participant is making up the pain), hostility (aggression such as bullying or teasing), and psychogenic (pain is related to mental health).^20^ The literature needs to further define what pain dismissal is, as well as the consequences of such dismissal. One literature review seeking to explore the social context regarding pain dismissal identified three themes utilizing a method of meta-ethnography of adults with chronic pain: stigma, experience of isolation, and experience of emotional distress^22^. Newton and colleagues found that having one’s pain disbelieved or attributed to psychological factors contributed to the stigma of having chronic pain. Wakefield and colleagues conceptualize pain dismissal in adolescents as an instance of “felt stigma”^18^. Participants described this felt stigma as feeling devalued as a result of others dismissing or minimizing their pain, others thinking that they were faking or exaggerating their pain, or attributing their pain symptoms to psychological factors. It is clear that forms of pain dismissal are related to feeling devalued, thus the psychological consequences of pain dismissal in EAs needs to be further explored.

In general, EAs that experience chronic pain are more likely to be described as isolated and sensitive, as well as less popular among their peers^23^. Furthermore, EAs who experience pain dismissal feel more isolated than those that do not experience pain dismissal^20^, ^22^. Similarly, EAs with chronic pain may also experience an increased likelihood of anxiety and depression^5^, ^24^. It is likely that having one’s pain dismissed may also lead to higher rates of anxiety and depression given the importance of validation towards those who experience pain. However, the effect of pain dismissal on anxiety and depression is unexplored in the literature. It is important to explore research questions within the context of a diverse sample, given the higher rates as well as the impact of pain stigma among minoritized groups^19^.

The current study aims to describe the lived experiences of emerging adults with chronic pain who have experienced pain dismissal, and the outcomes related to that dismissal. Currently research provides limited insight regarding chronic pain in EAs, the prevalence of pain dismissal, and the impact of experiencing dismissal. As such, individuals may not be receiving the appropriate health care for their pain, which may lead to further pain, worsening physical symptoms, mental health consequences, and increased functional disability. This study explored: 1) who experiences pain dismissal, 2) who is dismissing the pain of EAs, 3) what is experienced as pain dismissal, and 4) what is the impact of pain dismissal. Building on past research, it is expected that 40 % of EAs with chronic pain will experience pain dismissal. We will also build upon past research to examine pain dismissal among those identifying as another gender identity and across EAs of different racial and ethnic backgrounds. It is hypothesized that those with marginalized identities (other gender identity/those that are gender nonconforming, and non-White or Hispanic EAs) will experience greater pain dismissal. Second, given past research that caregivers and physicians tend to engage in pain dismissal, it is hypothesized that those that will engage in pain dismissal are caregivers/parents, medical providers, family members such as siblings, instructors, and peers. Third, pain dismissal is hypothesized to be experienced by these EAs in the forms of denial of pain, blaming of mental health, minimizing pain, normalizing the pain, refusal to help or refer out for treatment, and rationalizing. Fourth, building upon past research on the impact of pain dismissal in adults, it is hypothesized that pain dismissal will elicit negative emotional reactions, and dismissal will be associated with greater anxiety and depression. With this research, we aim to improve our understanding of what pain dismissal looks like and feels like for EAs with chronic pain.

## 2 Method

### 2.1 Procedure and Participants

Participants were students between the ages of 18–25 enrolled in introductory psychology courses at a Midwestern University during the 2023–2024 academic year. Those electing to participate completed study materials online via a Qualtrics survey and received course credit for their involvement via the SONA research system. Participation was estimated to take no more than 30 min. From the larger sample that completed the survey, only participants who reported experiencing chronic pain (pain that has lasted three months or longer) were included in this study (*N* = 227)

### 2.2 Measures

#### 2.2.1 Demographic information

Participants reported their age, sex assigned at birth, gender identity, ethnic and racial identity.

#### 2.2.2 Pain and pain dismissal questions

For pain over the past two weeks, participants reported the frequency of pain, pain location, duration, and pain intensity. Pain frequency was a forced choice question with number of days with pain ranging from 0 to 14. Pain location and pain duration were reported as open-ended responses. Pain intensity, both usual and worst pain intensity, was rated on an 11-point numeric rating scale ranging from 0 to 10 which is a reliable measure of pain intensity^25^. Participants were only included in the study if they reported having chronic pain, which was defined as pain duration of three months or longer.

Pain dismissal questions included “Thinking about the longest lasting pain problem, has a medical professional or someone close to you ever ignored or not believed you about your pain?”; and if yes, participants answered “Who dismissed your pain?”, “What was said?”, “How did it make you feel to have your pain ignored or not believed?”, and “How much did it bother you to have your pain ignored or not believed? (0 = not bothered at all and 10 = extremely bothered/upset).” These questions have been used in previously published papers on pain dismissal^20^.

#### 2.2.3 Anxiety

Participants reported anxiety symptoms on the GAD-7^26^. Seven items were presented as statements that were rated on a Likert Scale of 0–3; 0 (“not at all”), 1 (“several days”), 2 (“more than half the days”), 3 (“nearly every day”). Some sample items in this measure included “feeling nervous, anxious or on edge”, “not being able to stop or control worrying”, and “worrying too much about different things.” The sum of all the items was calculated; the possible range of scores was 0–21. The GAD-7 measure was previously validated within the EA age group^27^. The internal consistency of the GAD-7 was excellent, and test-retest reliability was also good, along with procedural validity.

#### 2.2.4 Depression

Participants reported depressive symptoms on the PROMIS Short Form - Depression^28^. Eight items were presented as statements that were rated on a Likert Scale of 0–5; 1 is “never”, 2 is “rarely”, 3 is “sometimes”, 4 is “often”, and 5 is “always”. Some sample items in this measure included “I felt worthless…”, “I felt depressed…”, and “I felt like a failure…” The PROMIS measure was validated within the EA age group^29^. Reliability and validity were good within the measure.

### 2.3 Data analyses

First, we examined the distribution of experiences of pain dismissal across emerging adults with chronic pain. We used chi-square analyses to determine whether there were differences in pain dismissal across demographic groups. Given the numerous demographic identities included, we collapsed gender categories into Man or Boy (n = 29, 12.8 %), Woman or Girl (n = 177, 78 %), and Other Gender Identity (n = 20, 8.8 %). We used the following ethnic categories for the chi-square analysis: European American (n = 96, 42.3 %), African American (n = 18, 7.9 %), Asian American (n = 26, 11.5 %), Latinx (n = 40, 17.6 %), and Other/Multiracial (n = 47, 20.7 %). To investigate the outcomes of the pain dismissal, we examined the range and mean scores of how “bothered” individuals were regarding pain dismissal. Finally, we used t-tests to determine whether participants who were dismissed experienced higher anxiety and depression than those not dismissed.

#### 2.3.1 Qualitative data

Secondly, we looked at open-ended questions and used thematic analysis to identify patterns and themes in the responses and code the responses to understand the feelings associated with pain dismissal. Thematic analysis, as described by Braun & Clarke, involves an iterative process of members of a team independently reviewing responses, identifying themes that emerge from the qualitative data, coming together to reach consensus on themes and definitions of themes, and recoding the data based on those themes^30^. Responses may have included content relevant to numerous themes, and therefore individual responses could be coded with multiple themes. This qualitative analysis was conducted for three open ended responses: what participants report was said that constituted pain dismissal, who dismissed the pain, and how it felt. The team did not use themes previously identified by Defenderfer; rather, the team independently generated codes based on the current study’s data. Three graduate student team members supervised by a doctoral level researcher engaged in this process and consensus was achieved by group discussion for each participant response.

## 3 Results

### 3.1 Descriptive statistics

Participants who reported experiencing chronic pain (pain that has lasted three months or longer) were included in this study (*N* = 227). Some survey respondents were excluded from the study for not having chronic pain (*n* = 452). Participants were between the ages of 18–25 (*M*age = 19.58; *SD* = 1.43). Participants were primarily European American (42 %), Other/Multiracial (21 %), or Latinx (18 %). Most were female (86 %) and identified as woman/girl (78 %). Detailed sex, gender, and race and ethnicity data is available in Table 1.

**Table 1:** Participant demographics.

	n(%)^a^
Sex	
Female	195(85.9)
Male	30(13.2)
Other	2(0.9)
Gender^b^	
Woman/Girl	177(78.0)
Man/Boy	29(12.8)
Nonbinary	5(2.2)
Gender Non-Conforming	5(2.2)
Gender Queer	3(1.3)
Genderfluid	2(0.9)
Other	5(2.2)
Race and Ethnicity	
European American	96(42.3)
Lantinx	40(17.6)
Mexican American	36(15.9)
Central or South American	15(6.6)
Cuban American	3(1.3)
Puerto Rican	1(0.4)
Other Latinx Nationality	1(0.4)
Mexican/Puerto Rican	5(2.2)
Mexican/Central or South American	1(0.4)
Mexican/2 + Latinx Identities	1(0.4)
Multiple Non-Mexican Latinx Identities	2(0.9)
Asian American	26(11.5)
Philippine American	7(3.1)
Indian American	6(2.6)
Chinese American	5(2.2)
Other Asian Nationality	5(2.2)
Pakistani American	4(1.8)
Korean American	2(0.9)
Japanese American	1(0.4)
Chinese/Other Asian Nationality	4(1.8)
Chinese/Korean American	2(0.9)
Chinese/Philippine American	1(0.4)
Multiple Non-Chinese Identities	1(0.4)
African American	18(7.9)
Other Singular Identity (e.g., Native American, Middle Eastern)	3(1.3)
Latinx/European American	18(7.9)
African American/European American	10(4.4)
Asian American/European American	8(3.5)
African American/Latinx	2(0.9)
Latinx/Asian American	3(1.3)
Other Multiracial/Ethnic Identity	3(1.3)

a % of 227 participants.

b One participant responded to the question about gender stating “I do not know what this question means.”

### 3.2 Pain characteristics

Pain duration ranged from 3 months to 240 months (n = 178, M = 48.26, SD = 43.36). Twenty-three people did not provide a duration of the pain problem, and five people reported the pain was lifelong. Over the past two weeks, participants reported an average of 7.15 pain days (SD = 3.92). The most common pain locations were multiple locations (n = 161, 70.9 %), back (n = 22, 9.7 %), stomach/abdomen (n = 17, 7.5 %), head (n = 15, 6.6 %), and other (n = 12, 5.3 %). Mean usual pain intensity was 3.30 (SD = 1.77) and mean worst pain intensity was 5.46 (SD = 2.30).

### 3.3 Pain dismissal and participant characteristics

Over 40 % of participants with chronic pain experienced pain dismissal (*n* = 98, 43 %) Pain dismissal was reported more frequently in women (48 %) and those identifying as another gender (50 %) compared to men (13 %; *X* ^2^ (2) = 12.59, *p* = .002). There was no significant difference in pain dismissal experiences between participants from different racial groups (i.e., European American, African American, Asian American, Latinx, and Other/Multiracial; *X* ^2^ (4) = 3.19, *p* = .53). Participants who reported being dismissed reported significantly higher levels of worst pain compared to those who had not experienced dismissal (see Table 2; *t* = 2.33, *p* = .02). There was not a statistically significant difference for usual pain intensity (*t* = 1.37, *p* = .17).

**Table 2:** Pain, anxiety, and depression scores.

			Dismissed		Not Dismissed	
	Minimum	Maximum	Mean	SD	Mean	SD
Usual Pain Intensity	0	9	3.48	1.85	3.16	1.70
Worst Pain Intensity	0	10	5.87	2.23	5.15	2.31
Anxiety	0	21	12.87	5.74	11.25	5.66
Depression	38.20	75.00	59.27	7.55	56.37	8.61

### 3.4 Pain dismissal experiences

Participants who experienced pain dismissal (*N* = 98, 43 %) responded to open-ended questions regarding who dismissed their pain, what was said to dismiss their pain, and how it felt to have their pain dismissed. In total, 96 % (*n* = 94) of participants who were dismissed provided interpretable and clear responses to who dismissed their pain, 96 % (*n* = 94) for what was said to dismiss their pain, and 97 % (*n* = 95) how it felt to have their pain dismissed. Primary themes were established for each qualitative question.

#### 3.4.1 Who dismissed pain?

Five themes were established to summarize responses to who dismissed participant pain (Table 3). Across 92 responses, a total of 114 instances of these themes were present. Of these 114 themes, *medical professionals* (*n* = 52, 46 %) and *caregivers/parents* (*n* = 43, 38 %) were most frequently reported people to have dismissed participant pain. Participants provided answers such as “Primary care doctor (20-year-old Mixed African American and Latinx woman),” “My mom, (22-year-old Mixed African American and Latinx woman),” and in some cases reported both *medical professionals* and *caregivers/parents* as being dismissive, sharing “My mom and doctor said it's too small to do anything about (23-year-old European American woman).” *Other family* members were reported as dismissing pain (*n* = 10, 9 %), as were *instructors* (*n* = 6, 5 %) and *peers* (*n* = 3, 3 %).

**Table 3:** Qualitative summary of participant responses to “who dismissed your pain?”.

Theme	Definition	Example	n(%)^a^
Medical professionals	Medical professional such as a nurse, primary care provider, or therapist.	“Primary healthcare provider, dermatologist, other medical professionals”	52 (45.61 %)
Caregiver/parents	Parent, guardian, or caregiver.	“My mother”	43(37.77 %)
Other family	Familial relationships other than caregivers/parents.	“My aunt”	10 (8.77 %)
Instructors	Individuals who hold the role of teaching, instructing, advising, or coaching.	“A dance teacher”	6 (5.26 %)
Peers	Friends, teammates, classmates, romantic partners, etc.	“My close friends"	3 (2.63 %)

a % of 114 total themes recorded across 92 participant responses.

#### 3.4.2 What was said?

Five themes were established to summarize responses to what was said to dismiss participant pain (Table 4). Across 97 responses, a total of 94 responses were able to be coded. Among these 94 responses, 113 instances of these themes were present. Of these 113 themes present, *minimizing/invalidating* (*n* = 31, 27 %) and *normalizing* (*n* = 29, 26 %) were most frequently reported. Participants provided responses detailing individuals *minimizing/invalidating* their pain with statements such as “i was being dramatic (21-year-old Indigenous woman)” and “Its not that bad (19-year-old European American woman)”. Responses indicating others normalizing their pain described it as being a typical part of life with statements such as “it's just a headache, take an aspirin (19-year-old European American nonbinary individual)” and “It's normal for women (19-year-old African American woman).” *Disbelief* of pain was noted frequently (*n* = 24, 21 %) in responses such as “It was in my head and not real (18-year-old Latinx woman)” and “I am faking it to get out of school (19-year-old European American nonbinary individual).” Others experienced *rationalizing* (*n* = 23, 20 %), stating “it was because of the things I ate (18-year-old Latinx woman),” “they said it was anxiety (19-year-old European American woman),” and “maybe you have slept in the wrong position don't think about it (19-year-old Asian American woman).” A smaller portion of individuals notably reported responses coded as *we can’t do anything* (*n* = 6, 5 %). These responses included “They did tests and they all came out normal, so the resulting response was basically a shrug and ‘go somewhere else (19-year-old European American nonbinary individual)’” and being told “…that it will go away soon and they can't do anything about it now (18-year-old European American woman)”.

**Table 4:** Qualitative summary of participant responses to “what was said?”.

Theme	Definition	Example	n(%)^a^
Minimizing/ invalidating	Suggestion that pain is not as severe as individual stated, suggestion that individual was being dramatic.	“that i was being dramatic”	31 (27.43 %)
Normalizing	Asserting that everyone has pain and it will go away with time or is nothing to worry about.	“its just a headache, take an aspirin”	29 (25.67 %)
Disbelief	Expressing that pain cannot be occurring, either due to making pain up or it being all in individual’s head.	“She thought I was using it as an excuse to not complete chores or was "selectively" having headaches.”	24 (21.24 %)
Rationalizing	Place blame or explaining cause of pain as a result of individual’s behaviors or specific mental health cause.	“probably a cold, etc.”	23 (20.35 %)
“We can't do anything”	Stating that there is nothing medical or otherwise that can be done to help with pain, refusing to refer to specialist care, or that the only option is to wait and see how pain progresses.	“… they don't know what's wrong with me and leave it at that.”	6 (5.31 %)

a % of 113 total themes recorded across 94 responses.

### 3.5 Emotional and psychological responses to dismissal

#### 3.5.1 How did it feel?

Eight themes were established to summarize responses to how it felt to have pain dismissed (Table 5). Across 94 responses, 89 responses had enough information to be coded. Across these 89 responses, 129 instances of these themes were present. Most frequently reported was a *generic negative feeling* (*n* = 32, 25 %) which included broad negative descriptions like “bad (19-year-old European American woman),” “all the negative things a 10 year old can feel. I don't wanna talk about it (22-year-old Asian American woman),” and “Like shit… (19-year-old European American nonbinary individual).” *Anger/Annoyed* (*n* = 27, 21 %) was the second most often theme reported. Participants detailed *anger/annoyed* emotional responses by saying “it made me angry… (19-year-old European American woman)” and “frustrated (20-year-old Mixed African American and Latinx woman).” *Negative self-view* (*n* = 20, 16 %) was characterized by statements related to self-worth and self-perception including responses like “i felt crazy and that i was looking for attention (21-year-old Indigenous woman)” and “I felt like maybe I was just being overdramatic (19-year-old European American woman).” Additional themes present were *invalidated/gaslit* (*n* = 16, 12 %), *socially negative* (*n* = 12, 9 %), *upset* (*n* = 10, 8 %), *sadness* (*n* = 7, 5 %), and *fearful/paranoid* (*n* = 5, 4 %). See examples of these and definitions in Table 5.

**Table 5:** Qualitative summary of participant responses to “how did it feel?”.

Theme	Definition	Example	n(%)^a^
Generic negative feeling	Expressing feeling bad or the like without specificity, numb, withdrawn, and/or lack of reaction, and feeling depressed and/or hopeless.	“Bad”	32 (24.81)
Anger/Annoyed	Expressing feeling angry, annoyed, irritated, frustrated, and the like.	“frustrating because this didn't go away and I just wanted to know how to help it feel better”	27 (20.93)
Negative self-view^b^	Expressing a personal critique of character, self-blame, or diminished self-worth.	“It just made me feel I was overreacting about the pain I was feeling”	20 (15.50)
Invalidated/Gaslit^b^	Expressing a sense of worthlessness or self-questioning because of what other people stated.	“It felt like my feelings weren't worth anything.”	16 (12.40 %)
Socially negative	Expressing feeling neglected, ignored, betrayed, disregarded, and/or mistreated by others.	“not seen.”	12 (9.30 %)
Upset	Explicitly stating feeling upset.	“Very upset”	10 (7.75 %)
Sadness	Explicitly stating feeling sad.	“Sad”	7 (5.43 %)
Fearful/Paranoid	Explicitly stating feeling fear, paranoia, worry and/or anxiety.	“I feared being removed from dances or losing my progress”	5 (3.88 %)

a % of 129 reported themes across 89 responses

b While similar *invalidated/gaslit* and *negative self-view* are distinct themes. *Invalidated/gaslit* responses emphasized feeling others viewed their feelings or experience as less worthy, valid, or meaningful while *negative self-view* emphasized personal critique and self-blame.

### 3.6 Survey results regarding psychological responses to dismissal

Mean scores of “bother” reported by individuals who were dismissed suggest that pain dismissal was bothersome (*M* = 6.78, *SD* = 2.33, *n* = 96; 0 = not bothered at all and 10 = extremely bothered/upset). Individuals who were dismissed reported higher anxiety compared to those who were not dismissed (see Table 2; *t* = 2.12, *p* = .04). Similarly, those who were dismissed also reported higher depression t-scores compared to those who were not dismissed (see Table 2; *t* = 2.65, *p* = .01).

## 4 Discussion

The goal of this paper was to explore the lived experiences of EAs with chronic pain who have experienced pain dismissal. This research aims to deepen the understanding of what pain dismissal entails, who is dismissing pain, and the emotional toll it can create. This study is important as EAs are in a developmental phase where they are gaining independence and establishing social and economic identities for those with chronic pain, these typical challenges are heightened by their health challenges. This study bridges the gap by specifically focusing on EAs and highlighting the social and emotional impacts of pain dismissal.

One goal of our study was to replicate and extend the research done on pain dismissal in EAs. On the whole, our results mirrored previous research regarding which EAs are dismissed, who did the dismissing, and what was said^20^. It is worth noting that our findings were consistent with previous research on the topic, even though our sample was more diverse than previous samples, and our data were collected several years later, after the onset of the COVID-19 pandemic. Those that were found to be dismissed most often included females, which is consistent with the findings of Defenderfer and colleagues^20^. Notably, we found that those of other genders/gender nonconforming individuals were also dismissed more often than males. Previous literature has not included many gender non-conforming folks. Including this vulnerable population is vital to understanding the relationship between pain dismissal, various layers of stigma, and gender-based discrimination. Contrary to our hypotheses, we did not find higher rates of pain dismissal in minoritized racial/ethnic groups. Racialized groups tended to report similarly high levels of pain dismissal (∼45 %); however, given possible lived experience with dismissal and discrimination from healthcare providers, it is possible that EAs carefully select to whom they disclose their pain. Due to the known additive layers of stigma experienced by EAs with pain and marginalized identities, this is an area that requires greater research^19^.

Those that commonly dismissed pain included caregivers and medical professionals, which is consistent with the findings of Defenderfer and colleagues. Researchers have found that those who contribute to pain dismissal often include caregivers and medical professionals, as well as immediate and extended family members, and even school staff^20^.

Emerging adulthood is characterized as a critical period of increased independence overall, including healthcare decision making, and includes the transition from pediatric to adult care. Medical professionals who dismiss pain during this transitional phase may also disrupt the process, leading to long-term consequences for healthcare engagement, trust, and management. The findings suggest that when EAs experience pain dismissal, particularly from medical professionals, it led to some of them questioning the validity of their own pain *“I started overthinking it and maybe considered that I was lying to myself*… (22-year-old European American woman)” or even felt like they were “crazy”. Several EAs also stated that their age and condition was also used to justify dismissing their pain “*I was told scoliosis doesn’t cause pain* (19-year-old European American woman)*”, “They said I was ‘too young* (20-year-old African American woman)*’”*. This is especially problematic during a phase where individuals are expected to manage their own care and make independent health decisions that rely on EAs feeling supported and confident. With that, experiences of dismissal, particularly from medical professionals, may create more barriers and increase the difficulty of the transition.

Overall, the most described forms of pain dismissal found in our study were *minimizing/invalidating* and *normalizing.* We also had several EAs report that the person dismissing their pain reacted with *disbelief;* however, this response was less frequent than in Defenderfer’s sample of EAs. Defenderfer and colleagues found that the most described form of pain dismissal was denial or that EAs were making it up^20^. Notably, many of the themes that emerged in this study overlapped considerably with previous findings (*minimizing, disbelief, rationalizing/psychogenic reasoning*)^18^. While there are many reactions and phrasings that can be experienced as dismissive of one’s symptoms, there is some consistency in the most common forms of pain dismissal. These responses may be unique to EAs for having pain due to their young age compared to adult samples. Newton and colleagues found themes reflecting more emotional reactions to pain dismissal in adults (distress, isolation), thus the emotional impact of dismissal is another important area of research that we aimed to address^22^.

A second goal of our study was to understand the emotional and psychological impact that pain dismissal has on EAs. The results of this study demonstrate that pain dismissal is generally a negative experience for most EAs with chronic pain. EAs with chronic pain who experienced pain dismissal also highlighted the negative emotional reactions and significant bother that are associated with this experience. The top feelings that pain dismissal invoked in these EAs were *generic negative feeling* and *anger/annoyed*. These results support the hypothesis that pain dismissal tends to bother individuals with chronic pain. This pain dismissal was also found to have a significant negative effect on mental health and overall well-being for EAs with chronic pain^24^. Emotional responses to pain dismissal also included *negative self-view* and *invalidated/gaslit* which both conveyed some negative views about oneself, including self-blame, self-doubt, and feelings of worthlessness. This is consistent with the literature on pain-related stigma in that individuals with pain may experience internalized stigma. This is when individuals with pain adopt the negative views of others and engage in self-blame, and feel embarrassed or ashamed of their pain^18^. Given that previous research suggested that pain dismissal is related to social isolation^7^, ^8^, this finding about the effect on one’s view of oneself is notable and troubling. It should be noted that loneliness and feelings of social isolation tend to peak during EA^31^, therefore social support should be a focus of evaluation and treatment of chronic pain in EA. Another notable finding of our study was that EAs with chronic pain that were dismissed reported higher rates of anxiety and depression than those that were not dismissed. These findings align with previous research showing that pain dismissal is linked to worse mental health outcomes^24^. Dismissing one’s pain experiences can contribute to depression through shame and stigma^22^, ^24^. Moreover, pain dismissal is related to higher rates of anxiety. This could be because individuals with chronic pain are already predisposed to anxiety symptoms, therefore they experience higher rates of anxiety when dismissed, since this negative social evaluation is an anxiety-provoking experience. Further, the results imply that dismissal not only from healthcare providers, but family and others can contribute to feelings of invalidation and isolation. This further highlights the importance of validation in chronic pain care, not just in healthcare settings, but at home and within day-to-day interactions.

### 4.1 Clinical implications

As it relates to interventions and pain management, interventions and patient education should be centered to increase empathy and validation in providers, caregivers, and family. This could help improve the mental health outcomes for those experiencing chronic pain. With that, further research should evaluate the impact empathy and validation have on emotional and physical outcomes for individuals with chronic pain. Given the negative impact of pain dismissal and the closeness of those who dismissed pain, it is important to educate providers, families, faculty, and staff on the importance of validating pain experiences. Additionally, patient-centered care approaches should be prioritized given the disproportionate dismissal of pain by medical professionals. Training health care professionals in empathic validation may be effective in changing individual providers’ behavior in validating patient symptoms^32^. Health care institutions would benefit from implementing formal transition related protocols that not only support the continuity of care for patients, but medical professionals as well^33^. Given the common themes of invalidation, disbelief, and rationalizing, EAs would also benefit from learning how to advocate when being dismissed, and having systems, resources, and supports in place for when they are dismissed.

### 4.2 Limitations

While our sample is more diverse with respect to gender, race, and ethnicity than previous studies, one limitation of the current study is that this university sample remains less representative of the demographics of typical EAs in the United States. The survey nature of the data collection did not allow for additional clarification to be asked of participants on qualitative responses. As such, some participants provided data that was unclear and thus uninterpretable. Future research should include more diverse samples to examine the different ways in which pain dismissal varies and the outcomes based on gender, cultural, and ethnic context, and offer participants additional opportunities to expand on their responses. Furthermore, studies should explore the long-term effects pain dismissal has on other chronic pain outcomes suggested by our findings, including care-seeking behaviors and social well-being.

### 4.3 Conclusion

As EAs navigate the significant lifestyle changes and challenges associated with the transition to adult health care, those experiencing chronic pain are further negatively impacted by the dismissal of their pain. Not only is chronic pain an added stressor, but the additional impact of pain dismissal exacerbates their existing challenges. As a result, this could consequently lead those impacted to have a bleak outlook on both their pain and future well-being. Interventions to change the response to EA’s experiences with pain may have physical and psychological benefits for EAs with pain.

## CRediT authorship contribution statement

**Elizabeth Fenelon:** Writing – review & editing, Writing – original draft, Visualization, Validation, Software, Resources, Project administration, Methodology, Investigation, Funding acquisition, Formal analysis, Data curation, Conceptualization. **Kayla McCracken:** Writing – review & editing, Writing – original draft, Visualization, Validation, Software, Resources, Project administration, Methodology, Investigation, Funding acquisition, Formal analysis, Data curation, Conceptualization. **Keely Bieniak-Fortier:** Writing – review & editing, Writing – original draft, Visualization, Validation, Software, Resources, Project administration, Methodology, Investigation, Funding acquisition, Formal analysis, Data curation, Conceptualization. **Chloe Crosby:** Writing – review & editing, Writing – original draft, Visualization, Validation, Resources, Methodology, Investigation, Formal analysis. **Paulina Paredes Cienega:** Visualization, Validation, Software, Resources, Project administration, Methodology, Investigation, Data curation, Conceptualization. Susan T. Tran: Writing – review & editing, Writing – original draft, Visualization, Validation, Supervision, Software, Resources, Project administration, Methodology, Investigation, Funding acquisition, Formal analysis, Data curation, Conceptualization.

## Funding statement

10.13039/100008093DePaul University provided financial support for the preparation of the article. A University Research Council Summer Research Grant funded three graduate students and one principal investigator for their work on this project.

## Declaration of Competing Interest

The authors declare that they have no known competing financial interests or personal relationships that could have appeared to influence the work reported in this paper.

## Data Availability

Data will be made available on request.
